# Core and periphery structures in protein interaction networks

**DOI:** 10.1186/1471-2105-10-S4-S8

**Published:** 2009-04-29

**Authors:** Feng Luo, Bo Li, Xiu-Feng Wan, Richard H Scheuermann

**Affiliations:** 1 School of Computing, Clemson University, Clemson, SC, USA; 2 School of Biology, Georgia Institute of Technology, Atlanta, GA, USA; 3 Department of Pathology, Division of Biomedical Informatics, U.T. Southwestern Medical Center at Dallas, Dallas, TX, USA

## Abstract

**Background:**

Characterizing the structural properties of protein interaction networks will help illuminate the organizational and functional relationships among elements in biological systems.

**Results:**

In this paper, we present a systematic exploration of the core/periphery structures in protein interaction networks (PINs). First, the concepts of cores and peripheries in PINs are defined. Then, computational methods are proposed to identify two types of cores, k-plex cores and star cores, from PINs. Application of these methods to a yeast protein interaction network has identified 110 k-plex cores and 109 star cores. We find that the k-plex cores consist of either "party" proteins, "date" proteins, or both. We also reveal that there are two classes of 1-peripheral proteins: "party" peripheries, which are more likely to be part of protein complex, and "connector" peripheries, which are more likely connected to different proteins or protein complexes. Our results also show that, besides connectivity, other variations in structural properties are related to the variation in biological properties. Furthermore, the negative correlation between evolutionary rate and connectivity are shown toysis. Moreover, the core/periphery structures help to reveal the existence of multiple levels of protein expression dynamics.

**Conclusion:**

Our results show that both the structure and connectivity can be used to characterize topological properties in protein interaction networks, illuminating the functional organization of cellular systems.

## Background

Network biology [1], which models biological systems as networks of connected elements, enables biologists to understand both macroscopic properties of biological systems [2-5] and microscopic properties of single molecules within systems [6]. With the advances in high-throughput techniques, more and more large-scale biological networks have been defined [7,8]. Studying the structure of biological networks will help elucidate the organization and functional relationships of elements in cellular systems.

Recently, Guimera et al. [9] classified the roles of nodes in complex networks according to their properties inside sub-network "modules". Their classification depended on dissecting the network into modules using a simulated annealing method [10]. However, precisely identifying biologically-relevant modules from PINs is not a trivial task. Fortunato and Barthelemy [11] recently pointed out that the optimization of Newman-Girvan modularity appears to favor large modules, and thus may miss important biological relationships that exist at the molecular level. Application of the method of Guimera and Amaral [12] to separate the yeast PIN from MIPS [13] into modules showed that these structurally-defined modules did not show a significant correlation with biological functional units. Thus, defining roles of proteins based on these modules may not be appropriate for PINs. However, it is still possible to understand the roles of proteins in the PINs within other types of sub-graph structures. In this study, we explore the role of proteins in PIN based on core/periphery structures.

**Figure 1:**
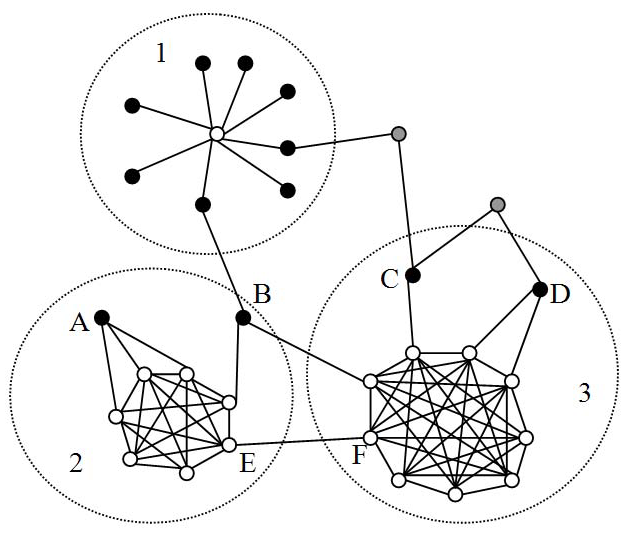
**A sample network including three core/periphery structures, which are denoted by the gray circles (1–3)**. The empty cycle nodes are core members. The black and grey nodes represent 1-peripheries and 2-peripheries, respectively. Labelled nodes (A-F) are different types of 1-peripheries: A) the closed single-core periphery; B) the multiple-core periphery; C) the complete-open single-core periphery; D) the limited-open single-core periphery; both E) and F) the core-member periphery.

Many studies have focused on the highly connected sub-networks in PINs [14-17]. However, most of these approaches exclude peripheral proteins that only connect to the core proteins with a few links, even though these peripheries may represent true interactions that have been experimentally verified [18]. The concept of core/periphery structures has a long history in social network analysis [19]. It wasn't until recently that the model of core/periphery structure in a network was first formalized by Borgatti and Everett [20]. In their model, a network contains a core/periphery structure if it can be divided into a core set, in which members are cohesively connected to each other, and a periphery set, in which members are loosely connected to the core members. Figure 1 shows an example network with three cores. The star (structure #1) is a special core/periphery structure with only one core member.

Core/periphery structures can be related to protein complexes. Protein complexes often include a static part in which components stably interact with each other all the time and a dynamic part that is assembled in a just-in-time fashion [21,22]. If the just-in-time assembled proteins only interact with small portion of the static part, the whole protein complex may appear as a core/periphery structure in the PINs. On the other hand, proteins that interact with different proteins in different contexts may emerge as a star structure in the PIN. Thus, the investigation of the core/periphery structure in PINs may help elucidate the dynamic of protein complex.

Furthermore, previous studies have shown that the structural characteristics, like connectivity (number of links), of proteins in PINs is related to the biological properties, such as essentiality [6] and evolutionary rate [23,24]. On the other hand, the roles and properties of proteins are also found to be related to the structural characteristics of proteins in the PIN [22]. However, the relationship between structural and biological properties of core and peripheral proteins in PIN has not been fully explored. It is plausible to hypothesize that the core and peripheral proteins may have different roles and properties due to their different topological characteristics. For example, core proteins are usually more highly connected to each other and may have higher essentiality characteristics and lower evolutionary rates than those of peripheral proteins. Combining the structural characteristics of proteins with their biological properties may help elucidate their different roles in biology systems.

In this paper, we present a systematic exploration of core/periphery structures in PINs. Our studies help elucidate the relationship between topological properties in PINs and the roles played by proteins in cellular system, and thus help define the organizational mechanisms used in cellular system.

## Core/periphery structures in PINs

A PIN can be modeled as an undirected and unweighted graph G = (V, E), where the vertices set V represents proteins and the edges set E represents interactions between proteins. In the context of this paper, the graph is synonymous with the network. A core [25] in a network is a cohesive sub-graph, in which nodes are highly connected to each other. There are various definitions of cohesive sub-graph based on different connectivity properties of the vertices, including cliques [26], k-plexes [27], k-cores [28] and n-cliques [29].

A clique is a complete sub-graph of three or more nodes in which all nodes are connected to each other. A maximum clique is a complete sub-graph in the graph such that there are no nodes remaining in the graph that are connected to all the member of the clique. However, the clique is a very restrictive sub-group definition for protein interaction networks. Two concepts in network theory have been proposed to loosen the clique definition. The n-clique relaxes the requirement on the distance between nodes inside the sub-graph. An n-clique is a sub-graph in which all pairs of nodes are no greater than n distance apart. Unfortunately, the n-clique are often not be very cohesive, even for a 2-clique. The k-plex sub-graph definition relaxes the number of nodes required to be connected for each node in the sub-graph. A k-plex is a sub-graph in which each node is connected to at least n-k nodes, where n is the number of nodes in the sub-graph and k is a tunable parameter. Another cohesive sub-graph is the k-core. A k-core is a connected maximal sub-graph in which each node has degree (number of connections) at least k. Although the k-core includes all cohesive sub-graphs, it may also contain non-cohesive parts. Another problem with the k-core approach is that k-cores cannot overlap. Based on these considerations, we expect that the k-plex approach will likely provide a better representation of functionally relevant sub-graph cores in protein interaction networks.

In this study, we define a core in a PIN as a local maximal k-plex with k ≤ n/2 , where n is the number of nodes in the sub-graph. The local maximum means that no more peripheral node can be added into the sub-graph such that the sub-graph remains a k-plex at a given k.

We also define the k-periphery of a core as the set of nodes that are not in the core and whose distances to any member in the core are equal to k. For example, the 1-periphery is the set of nodes that are directly connected to core members (distance equals to 1). Our definition is different from the original definition of k-periphery by Everett and Borgatti [20], in which the k-periphery also includes nodes whose distances to any member of the core are less than k. Here, we will focus our study on the 1- and 2-peripheries of a core.

One special core/periphery structure is the star. In an ideal star, one single node is the core, and there are no connections between the peripheral nodes. For biological networks, we will allow limited connections between peripheral nodes, which will be controlled by the peripheral degree defined below.

### Types of 1-peripheral nodes

**Figure 2:**
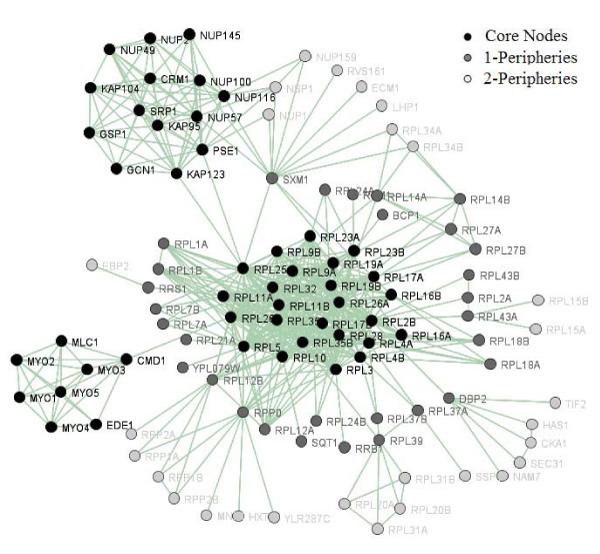
**The peripheries of the largest k-plex core in YPIN**. Among the 1-peripheries of largest k-plex core, CMD1, PSE1 and KAP123 are three core-member peripheries of this largest k-plex core. SXM1, DBP2, RPL43A are the multiple core peripheries of the largest kplex core. Graph is produced using Biolayout [47].

**Figure 3:**
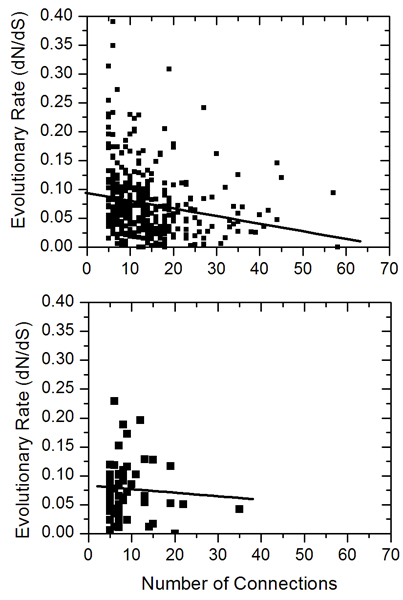
**The correlation between interactions and evolutionary rates of star core proteins and k-plex core proteins**. A) The correlation between interactions and evolutionary rates of k-plex core proteins. The correlation observed is -0.1934. B) The correlation between interactions and evolutionary rates of the star core proteins. The correlation observed is -0.0688.

Based on how they are connected to the core members, we classify 1-peripheral nodes into the following types: (1) the closed-single-core peripheral nodes (closed), which are only connected to members of one core (node A in Fig. 1); (2) The multiple-core peripheral nodes (multiple-core), which are connected to members of at least two different cores and may also be connected to other non-core nodes (node B in Fig. 1); (3) The open-single-core peripheral nodes (open), which are connected not only to members of one core but to other non-core nodes. This type of peripheral nodes can be further divided into complete-open-single-core peripheral nodes (complete-open), which have fewer connections to core members than to other non-core nodes (node C in Fig. 1), and limited-open-single-core peripheral nodes (limited-open), which have more connections to core members than to other non-core nodes (node D in Fig. 1); (4) The core-member peripheral nodes, which are members of one core and the 1-peripheries of some other cores (node E and F in Fig. 1). The delineation of these 1-peripheral node types will allow us to investigate if these structural distinctions have biological correlates.

### Structural measures for 1-peripheries

The characteristics of 1-peripheries can be described by the following structural measures:

#### Coreness (Cp)

Cp of a 1-periphery node is defined as the ratio of the number of its connections to the core members over the total number of core members, 0 < Cp < 1. The coreness measures the closeness between the 1-periphery node and the core members.

#### Participation Rate (Pr)

The Pr of a 1-periphery node is defined as the number of its connections to the core members over the total number of its connections. 0 < Pr ≤ 1. The Pr measures the level at which the 1-periphery members participate in the core. The Pr of closed single-core 1-periphery nodes is 1. The Pr of complete-open 1-periphery nodes are less than 0.5 and the Pr of limited-open 1-periphery nodes are greater than or equal to 0.5.

#### Peripheral Degree (Pd)

The Pd of a 1-periphery node is defined as the number of its connections to other 1-periphery nodes over the total number of peripheral nodes of the core [25]. 0 ≤ Pd < 1. The Pd measures the degree to which the 1-periphery nodes are connected to each other. Pd = 0 means that the 1-periphery node is only connected to the core members.

## Results

### Cores/peripheries are identified from the YPIN

Our KL-like algorithm has identified 110 k-plex cores with size of no less than six from the YPIN. Additional file 1[30] lists all proteins in obtained k-plex cores and their 1- and 2-peripheries. In total, 712 k-plex core proteins are identified. Some cores share a small overlap of their members. For example, cores 4 and 5 each have 16 "ubiquitin-dependent protein catabolic process" related proteins and share two protein members, PRE1 and RPN10. Most of the k-plex cores are part of protein complexes according to the MIPS protein complex database [13] (see Additional file 2[30]). Moreover, most k-plex cores contain all four types of 1-periphery nodes. Additional file 3[30] shows the details of the four types of 1-periphery nodes of each k-plex core. The largest k-plex core has 25 members. Figure 2 showed the largest k-plex core and its 1-, 2- peripheries. Then, we examined how the most significant GO terms among k-plex core members are ranked in the significant GO terms among their 1- and 2-peripheries using the gene ontology (GO) term finder from the Saccharomyces Genome Database (SGD) [31]. Additional file 4[30] shows that the significant GO terms in 92 k-plex cores are also significant in their 1-peripheries, but only 61 of them are significant in their 2-peripheries.  These results suggest that 1-periphery proteins are more related to core members' biological functions.

Based on the criteria for the star structure, we identified 109 star cores with at least five 1-periphery nodes from the YPIN. Additional file 5[30] lists all star core proteins and their 1-peripheries and 2-peripheries.

### Biological properties are different among cores and 1-peripheries

#### Comparing biological properties of k-plex core proteins and their 1-peripheries

First, we examined the average Pearson correlation coefficients (PCCs) based on five microarray data sets [32-36] using the same approach as Han et al. [22] (see Methods for details). Paired two-tail Student's T test showed that average PCC of k-plex core proteins are significant higher (p-value < 1.00E-3) than average PCC of all four types of 1-periperal proteins as shown in Table 1. Second, we tested the evolutionary rate [37] of the core proteins and 1-peripheral proteins. As shown in Table 1, the average evolutionary rate of core proteins is also significantly lower than the average evolutionary rate of complete-open (p-value = 3.19E-2), limited-open (p-value = 2.19E-4), closed (p-value = 1.63E-8) and multiple-core 1-peripheries (p-value = 1.97E-4). Third, we inspect the essentiality [38] of core proteins and 1-peripheral proteins. The Table 1 showed that the core proteins are much more essential than the open and closed 1-peripheiries. And paired two-tail T test also demonstrated the significant difference (p-value < 1.00E-5). Finally, we analyze the number of pfam protein domains in each protein, which is downloaded from SGD database [31]. The results (Table 1) showed that the average number of domains of core proteins is significantly greater than those of limited-open (p-value = 8.12E-6), closed 1-peripheries (p-value = 7.56E-7) and multiple-core 1-peripheries (p-value = 2.23E-2). However, there is no significant difference between the average number of domains of core proteins and those of complete-open (p-value = 1.23E-1) and between the average number of domains of core proteins.

**Table 1:** Comparison of properties of k-plex core members with those of different types of 1-peripheries.

	Average PCC	Evolutionary rate	Protein essentiality	Number of domains
k-plex core	0.2366	0.0770	0.4761	1.9968
Multiple-core peripheries	0.1293	0.0970	0.3082	1.7612
Complete open peripheries	0.1145	0.0885	0.3077	1.8018
Limited open peripheries	0.1646	0.1047	0.2377	1.5254
Closed peripheries	0.1222	0.1094	0.1930	1.4875
P value (core vs. complete open)	2.03E-14	0.0319	1.88E-6	1.26E-1
P value (core vs. limited open)	2.16E-5	2.19E-4	1.12E-11	8.12E-6
P value (core vs. closed)	3.52E-9	1.63E-8	3.62E-22	7.56E-7
P value (core vs. multiple)	1.09E-23	1.97E-4	3.79E-9	2.28E-2

Listed values are the average value of different properties of all members in each core and 1-periphery category. The p values are obtained by Student's T-test that is based on properties of all members of each core and 1-periphery category.

#### Comparing biological properties of star core proteins and their 1-peripheries

We compared 109 star cores (without 14 k-plex core members) with their 1-peripheries in four types of biological properties, including evolutionary rate, protein essentiality, number of domain, and average PCC. As shown in Table 2, except for the average PCC, there is no significant difference between star proteins and their 1-peripheries in the evolutionary rate, protein essentiality and number of domains. This indicates that, as biological process connectors, star core proteins are similar to proteins that they connect together.

**Table 2:** Comparison of properties of star cores with those of their 1- peripheries and k-plex cores.

	Average PCC	Evolutionary rate	Protein essentiality	Number of domains
Star cores	0.0838	0.0772	0.3303	1.7188
1-peripheries	0.1217	0.0906	0.2908	1.9379
P value (star vs. 1-p.)	1.03E-3	0.08	0.41	0.13
P value (star vs. k-plex)	8.54E-28	2.03E-4	0.0034	0.061

Listed values are the average properties in all members in 1-periphery. The p values are obtained by Student's T-test that is based on properties of all members of each core and 1-periphery category.

#### Comparing biological properties of star core proteins and k-plex core proteins

We compared the biological properties of star cores and those of k-plex cores. As shown in Table 2, except for number of domains, the average PCCs, evolutionary rate, and essentiality of k-plex core proteins are significantly different from those of star cores.

### The k-plex cores can consist of party proteins, date proteins, or both

In order to examine the differences among k-plex core proteins, we analyzed how the k-plex core members related to the date/party concepts of Han et al. [22]. Five microarray data sets [32-36] were used to determine the date and party classification of core proteins (see Methods for details). As a result (see Additional file 6), among the 706 proteins with PCCs in k-plex cores, 177 are party proteins and 529 are date proteins. Meanwhile, all star cores are date proteins except three. Moreover, lowering the threshold with significance level to 75% will not affect the conclusion that most k-plex core proteins are date proteins. This is a surprise, as the date core proteins are inside the functional modules (complex), rather than the external connectors [22]. The party and date core proteins have similar degree (average 13.435 vs. 13.762). The Student's t-test has shown no significant difference on degree distribution. However, the clustering coefficients [5] of party core proteins (0.5824) are significantly higher than those of date core proteins (0.4259). We then classified the k-plex cores according to the party and date proteins inside them. A party core consists entirely of party proteins. A date core, on the other hand, consists entirely ofdate proteins. A mix core will include both party and date proteins. There are only 7 party cores, 37 mix cores and 66 date cores (see Additional file 7). This classification implied that the formation and evolution of protein complexes may involve different mechanisms.

### The negative correlation between evolutionary rate and connectivity are much stronger among k-plex core members than among star core members

The relationship between evolutionary rate and connectivity of proteins has been investigated recently [24,39,40]. However, controversial results on the presence or absence of correlation between evolutionary rate and connectivity have been obtained. Here, we examined the relationship between evolutionary rate and connectivity in both the k-plex core and star core members. As the star cores have at least five connections, we also only examine the k-plex core proteins with at leas five connections. We observed a negative correlation -0.1934 within k-plex core members (Figure 3A).  However, very weak correlation has been observed within star core members (correlation is -0.0688, Figure 3B). The combination of k-plex core and star core members also showed a negative correlation of -0.1828. These results suggest that the negative correlation between evolutionary rate and connectivity comes from the k-plex core members only, but not from the star cores. This detailed analysis showed that there are different evolutionary rates patterns between k-plex cores and star cores.

### Expression dynamics are different among different kinds of links connecting core proteins and 1-periphery proteins

To get further insight to the expression difference between k-plex core proteins and their 1-periphery proteins, we compared the average PCCs of microarray expressions between two core proteins and between one core protein and one 1-periphery protein (see Methods for detailed calculation). For each k-plex core, the Additional file 8[30] listed the average PCCs for links between k-plex core members and for links between k-plex core members and their 1-peripheries. The overall average PCC for links between k-plex core members is 0.2532. And the overall average PCC for links between k-plex core members and their 1-peripheries is 0.1799. Two-tail T test on the average PCCs between two kinds of links shows significant difference (p-value = 1.74E-3).

### Structural characteristics of 1-periphery proteins of k-plex cores imply two classes of 1-periphery proteins

The Additional file 9[30] lists values of three structural measures: Cp, Pr and Pd, of 1-peripheral proteins of each k-plex core. Table 3 compares the average values of these three different measures among different types of 1-periphery proteins. The average Cp over all five types of 1-periphery proteins is less than 0.2. This indicates that the 1-peripheries of cohesively connected cores in the PIN are far away from becoming members of cores, which implies that peripheral members of protein complex are only connected to a small part of the complex core.

**Table 3:** Average of three structural measures of 1-peripheries

1-peripheries type	Cp	Pd	Pr
limited-open	0.1646	0.0076	0.5458
complete-open	0.1285	0.0103	0.2601
closed single-core	0.1437	0.0000	1.0000
multiple-core	0.1529	0.0216	0.2378
core-member	0.1841	0.0724	0.1222

Furthermore, the Pd of all types of 1-periphery proteins are very small, 0.0724 for core-member 1-peripheries and less than 0.022 for other types of 1- peripheries. The low Pd of 1-peripheries indicates that 1-peripheries are generally not connected to each other. Thus, the peripheral members of the protein complexes usually may be assembled at different times and may involve distinct biological functions.

The Pr measures how the 1-periphery proteins connect to core members. All participation rates of closed single-core 1-periphery proteins are 1. The average participation rate of limited-open-single-core 1-periphery proteins is 0.5458. The average participation rates of complete-open, multiple-core, and core-member 1-periphery proteins are small, which indicates that the level that these three types of 1-peripheries associate with the cores is low. The high participation rates of closed and limited-open 1-periphery proteins indicate that they are more likely to join the protein complex (not the core). The low participation rates of multiple and complete-open 1-periphery proteins indicate that they are more likely to participate in different functionality or processes as they are connected to different complexes or individual proteins.

Therefore, we propose that closed and limited-open are "party periphery" proteins and multiple and complete-open are "connector periphery" proteins. As shown in  Figure 2, most closed and limited-open 1-periphery proteins of the largest k-plex core are also part of the ribosome complex. Furthermore, we compared four properties, evolutionary rate, protein essentiality, number of domain, and average PCC, between the party periphery proteins and connector periphery proteins. The student's t-test showed that the party periphery proteins are significantly different from connector periphery proteins in all four properties, number of domains (p-value = 3.59E-4), essentiality (p-value = 6.40E-5), average PCCs (p-value = 1.74E-2) and evolutionary rate (p-value = 9.27E-3).

### Both connectivity and topology are related to the property differences between k-plex core proteins and their 1-peripheral proteins

As the average connection (13.64 of k-plex core proteins are significantly greater than those of different types of 1-periphery proteins: closed-single-core (1.43), limited-open-single-core (3.13), complete-open-single-core (4.81) and multiple-core (6.38), it is worth examining whether the differences between the biological properties of the core proteins and those of 1-periphery proteins are associated with connectivity or topology differences. As shown in Table 4, only the average PCC of "party periphery" proteins are positively correlated with their connectivity (correlation > 0.28); the evolutionary rates of k-plex core proteins and "connector periphery" proteins are both negatively correlated with their connectivity (correlation < -0.15); only the essentiality of complete-open 1-periphery proteins are positively correlated with connectivity (correlation > 0.15). Thus, the connectivity difference of the nodes may be related to the difference in their biological properties.

**Table 4:** Correlation between connectivity and biological properties for different types of proteins

	Average PCC	Evolutionary rate	Protein essentiality	Number of domains
k-plex core	0.02625	-0.2059	0.1175	0.1349
Multiple-core peripheries	-0.05562	-0.1690	0.1155	0.0833
Complete open peripheries	-0.03379	-0.1585	0.1616	0.0375
Limited open peripheries	0.2852	-0.0965	0.0617	0.0018
Closed peripheries	0.2841	-0.0455	-0.0175	-0.1235

On the other hand, as shown in Table 4, there is no statistically significant correlation found in the following pair-wise comparisons: (1) between average PCC and connectivity for k-plex core proteins and "connector periphery" proteins; (2) between evolutionary rates and connectivity for "parity periphery" proteins; (3) between essentiality and the connectivity for all kinds of proteins rather than complete-open 1-periphery proteins; and (4) between number of domains and the connectivity for all kinds of proteins. Thus, in these cases, the topological types instead of connectivity may contribute to the difference in biological properties between nodes. In summary, both connectivity and topology are shown to be associated to the biological properties of proteins.

## Discussion

In this paper, we systematically explored the core/periphery structures in YPIN. We have identified 110 k-plex cores. Gene ontology based analysis showed that the 1-periphery proteins are closely related to the k-plex core proteins. However, low average coreness values of 1-periphery proteins indicated that peripheral proteins are structurally different from the k-plex core proteins. Furthermore, the properties of 1-peripheral proteins are significantly different from those of k-plex core proteins. Thus, it is meaningful to separate peripheral proteins from k-plex core proteins.

Based on their structural relationship with core members, we classified the non-core 1-periphery proteins into four types: closed-, limited-open, complete-open and multiple-core 1-periphery proteins. The closed and limited-open 1-periphery proteins, which have high participation rates, are structurally "party periphery" proteins. The complete-open and multiple-core 1-periphery proteins, which have low participation rates, are structurally "connector periphery" proteins. This classification may help understand different roles of 1-peripheiral proteins relate to the complex core. The "party periphery" 1-peripheral proteins are usually closely related to functionality of protein complex. On the other hand, the "connector periphery" 1-peripheral proteins are connectors that link the complex to other complexes or individual proteins.

Our results showed that the topological structures characteristics of proteins in PINs are reflected in their biological properties. For example, the closed and limited-open 1-periphery proteins have very similar topological structures and also have very similar biological properties. Furthermore, our results showed that, besides the connectivity, other structural characteristics are also related to biological properties. Thus, it is not enough to differentiate proteins based on connectivity only. Moreover, our studies showed that structure-properties relationship may be needed to take further analysis. For example, by further examining the relationship between the evolutionary rate and connectivity, we showed that there are differences between k-plex core proteins and star proteins.

The studies on the core/periphery structures in protein networks have also helped reveal expression dynamic difference in protein complexes [21,22,41]. The average PCC values of k-plex core members are significantly higher than those of their 1-peripheires. Furthermore, the average PCC values of links between k-plex core members are significantly higher than those of links between k-plex core members and their 1-periphery proteins. This dynamic difference implies the temporal "plug-and-play" components of protein complexes join the complexes after their formation.

## Methods

### Datasets

We have compiled a yeast PIN (YPIN) by combining three curated yeast PINs: "Filtered Yeast Interactome" (FYI) [22], the Structure Interaction Network (SIN) [42], and the yeast core PIN downloaded from the DIP database (version ScereCR20070707) [43]. After removal of all self-connecting links, the combined YPIN included 2,945 yeast proteins and 8,421 interactions. We applied our analysis to the single large component with 2,664 interconnected proteins (8,161 links) of this YPIN.

### Algorithm for identifying k-plex cores

Borgatti and Everett [20] developed a genetic algorithm to separate small social networks into one core and its periphery. However, Boyd et al. [44] found that the Bett algorithm does not give the optimal results in most test cases. Rather, Boyd et al. found that the Kernighan-Lin (KL) [45] algorithm performs better in partitioning social networks into a core set and a periphery set. Here, we adapt the KL algorithm to identify all k-plex cores in the PINs.

The KL algorithm takes a heuristic approach to find a locally optimal partition of a graph both effectively and efficiently. The essential idea behind the KL algorithm is the designation of the gains associated with moving a node between two different sets. Thus, the problem of finding *k-plex *cores can be reduced to a local graph partitioning problem. We define the gain of moving a node into or out of a core set as following:

(1) $$gain(i)=\sum_{j\in Core}a_{ij}$$

where

(2) $$a_{ij}=\{\begin{array}{ll} +1 & if\;i\;and\;j\;are\;connected\\ -1 & if\;i\;and\;j\;are\;disconnected \end{array}$$

The rationale behind this is that we would like to favor edges between core members and penalize disconnections between core members. This gain-based approach will result in a k-plex (k ≤ ⌊*n*/2⌋) with all core members having positive scores.

The choice of value of the k parameter in the k-plex definition will affect the cohesion of the sub-network. If k is too small, the sub-network will be too cohesive and exclude some loosely connected core members. On the other hand, if k is too large, the sub-network will be too loose and include some peripheral nodes into the core. The choice of k ≤ ⌊*n*/2⌋ in our k-plex core definition was selected to balance these interrelationships such that each core member must connect to at least half of the members in the core.

Our KL-like algorithm will start from each node triangle in the PIN. Each cycle, the algorithm will move nodes from periphery to core or from core to periphery to create a new core. Then, the next moving cycle will continue with the new core and its periphery. This procedure will continue until no new cores can be generated. The KL-like algorithm employs a greedy moving mechanism. Every time, the algorithm will move one node with the maximal gain among nodes in the core and its periphery. If a new core with a higher gain is obtained after a move, it will be recorded.

The obtained k-plex cores will be refined by merging the cores sharing more than half of their members. It is noted that the refinement will yield some of members in the final cores that do not satisfied the k-plex requirement. The extended KL algorithm for identifying k-plex cores is summarized as follows:

1) For every triangle of nodes in the network

2)    Set triangle as the current best core set

3)    Do

4)       Set the current best core as current core set

5)       Set the current best core as previous best core

6)       For every node in current core set and its 1-periphery but not in initial triangle

7)          For every node in current core set and its 1-periphery but not in initial triangle

8)             If it has not been moved

9)                Calculate the gain of moving

10)             End-if

11)          End-for

12)          Move the node with *best *gain

13)          If the score of the current core is higher than the current best core

14)             Store current core as the current best core

15)          End-if

16)       End-for

17)    While the current best core has a greater score than the previous best core

18) End-for

19) Prune the results and remove the replicate cores.

### Algorithm for identifying star structures

The procedure for locating star cores begins with finding all nodes that are not members of any k-plex core and have degree of at least five. Once these potential star nodes have been found, the periphery degrees of their 1-periphery nodes are examined. If the periphery degrees of all 1-peripheral nodes are greater than 0.16, the star node is kept. All star nodes that pass the examination are accepted as valid star cores. The reason that we choose 0.16 as the threshold for periphery degree is because it is possible to have stars with clustering coefficients greater than 0.1 if the maximum periphery degree threshold is beyond 0.16.

### Determination of party and date proteins

Five microarray data sets [32-36] were downloaded from the Yeast Functional Genomics Database (YFGdb). All five data sets have at least 50 experiment data points, which should ensure the accuracy of the calculation of PCC. For each data set, the average PCC of each protein is obtained by averaging the PCC between the proteins and their neighbour proteins. Unfortunately, plotting the probability distribution of average PCC showed no clear bimodal distribution for all five data sets. Similar observations have been obtained by Ekman et al. [46]. Instead of arbitrarily assigning a threshold, we modelled the distribution of average PCC as a normal distribution and calculated the mean and standard deviation of the distribution. We determined the threshold that separate "party" and "date" protein as the value that 90% of average PCC is below. Namely, the threshold for each microarray data set is the mean of average PCCs plus 1.282 times standard deviation. Addition file 6 lists the thresholds for all five microarray data sets. "Party" proteins are defined as proteins that have an average PCC from any of five microarray data sets higher than the threshold of that microarray data set; otherwise it is a "date" protein. Noted that we did not just classify hubs (with more than 5 links), but all core proteins.

### Calculation of average PCCs of proteins

For each data set, the average PCC of each protein is obtained by average the PCC between the proteins and its neighbour proteins. Then, the average PCC of a protein is calculated by averaging the five average values from the five data sets.

### Calculation of average PCC of links

For each data set, the PCC between each pair of connected proteins is calculated. The PCC of a link is the average PCC from the five data sets.

## Competing interests

The authors declare that they have no competing interests.

## Authors' contributions

FL developed the original ideas. FL and BL implemented the algorithms. FL, XFW and RHS: Analyzed the results and wrote the manuscript.

## Supplementary Material

Additional File 1 **Supplemental Table S1**. The k-plex cores in yeast protein interaction network and their 1- and 2-peripheries. Click here for file

Additional File 2 **Supplemental Table S2**. Participation of k-plex cores in MIPS protein complex. Click here for file

Additional File 3 **Supplemental Table S3**. Different types of 1-peripheries in k-plex cores. Click here for file

Additional File 4 **Supplemental Table S4**. Most significant GO terms of k-plex cores and their rank in peripheries. Click here for file

Additional File 5 **Supplemental Table S5**. Star cores in yeast protein interaction network and their peripheries. Click here for file

Additional File 6 **Supplemental Table S6**. Date and party hub classification of k-plex core and star core members. Click here for file

Additional File 7 **Supplemental Table S7**. Date and party classification of k-plex core. Click here for file

Additional File 8 **Supplemental Table S8**. Comparison of the average PCC between different types of links. Click here for file

Additional File 9 **Supplemental Table S9**. Structural properties of 1-peripheries of all k-plex cores. Click here for file
